# Nursing Students’ Experiences of Learning Evidence-Based Practice Through a Flipped Classroom: A Qualitative Study

**DOI:** 10.3390/nursrep16050149

**Published:** 2026-04-23

**Authors:** Verónica Pérez-Muñoz, Antonio Jesús Ramos-Morcillo, Alonso Molina-Rodríguez, María Ruzafa-Martínez

**Affiliations:** 1 Rafael Méndez University Hospital, 30800 Lorca, Spain; veronica.perez3@carm.es; 2 Murcian Health Service, 30100 Murcia, Spain; 3 Faculty of Nursing, University of Murcia, 30120 Murcia, Spain; alonso.molina@um.es (A.M.-R.); maruzafa@um.es (M.R.-M.)

**Keywords:** flipped classroom, evidence-based practice, nursing education, qualitative research

## Abstract

**Background:** Evidence-based practice (EBP) is a cornerstone of high-quality and safe nursing care. However, undergraduate nursing students often experience cognitive, methodological, and contextual barriers to learning and applying EBP. Active teaching strategies, such as the flipped classroom, may support the development of EBP competencies, yet qualitative evidence exploring students’ learning experiences remains limited. **Objectives:** To explore nursing students’ perceptions and experiences of learning evidence-based practice through a flipped classroom model. **Methods:** A qualitative descriptive study was conducted at the Faculty of Nursing of the University of Murcia (Spain). Purposeful maximum variation sampling was used to recruit undergraduate nursing students from the second and fourth academic years who had completed an EBP course delivered using a flipped classroom approach supported by an online learning platform. Twenty semi-structured interviews were conducted via videoconference. Data were transcribed verbatim and analyzed using reflexive thematic analysis with independent coding by two researchers and consensus procedures. Ethical approval and confidentiality were ensured. **Results:** Three main themes were identified: (1) transformation of the meaning of EBP learning and professional role, (2) cognitive and metacognitive processes in EBP learning, and (3) the learning experience as a catalyst for deep learning. Students described a shift from initial fear and perceived difficulty toward recognizing the practical value of EBP, accompanied by increased critical thinking, autonomous learning, and a growing evidence-informed professional identity. The flipped classroom model facilitated engagement and understanding, while the transfer of learning to clinical practice was influenced by contextual facilitators and barriers. **Conclusions:** Learning EBP through a flipped classroom was experienced as a transformative process that fostered critical thinking, self-regulated learning, and the construction of an evidence-oriented professional identity among nursing students. Strengthening information literacy skills and improving alignment between academic and clinical environments may enhance the sustainable application of EBP in clinical practice.

## 1. Introduction

Evidence-Based Practice (EBP) is a fundamental approach in modern healthcare, as it entails systematically integrating scientific evidence with patient needs, while considering the experience of healthcare professionals and the clinical context in which the intervention occurs [1,2]. The adoption of EBP in the health sector markedly enhances the quality of care, augments patient safety, optimizes health outcomes, diminishes healthcare expenses, and elevates the satisfaction of nursing professionals [3,4]. More recent literature has further highlighted the relevance of EBP in contemporary health care [5] particularly in relation to the context of the need for safe, up-to-date, and context-responsive clinical decision-making [6,7]. In recent years, EBP has evolved in the field of health and continues to consolidate as an essential component in the development of the nursing discipline [8,9].

Nursing degree training in this area, whether integrated across subjects or delivered through an EBP-specific course, enhances nursing students’ competency acquisition [10]. A recent meta-analysis concluded that EBP-centered training programs significantly improve the clinical competencies, critical thinking skills, and problem-solving abilities of nursing students [11]. Additionally, EBP-specific training and active supervision in clinical settings encourage students to apply EBP more frequently [12]. Despite apparent positive outcomes from EBP teaching, the recent literature shows that nursing students encounter significant cognitive and methodological obstacles to learning, especially in understanding research, conducting bibliographic searches, engaging in critical reading, and interpreting results [13]. These challenges are compounded by contextual and collaborative barriers in clinical environments, along with negative or reductionist views of EBP, often seen as not very useful or as solely focused on literature searches, without sufficiently incorporating clinical experience or patient preferences [14].

Lately, the flipped classroom approach has gained more attention. This pedagogical approach shifts the focus from instructors to students and aligns with constructivist theories and principles of adult education. It turns students into active participants in the construction of knowledge, rather than passive recipients of information [15,16,17]. The flipped classroom model is based on the prior delivery of theoretical content to students, allowing in-person class sessions to be dedicated to consolidating and enhancing learning through interactive, collaborative problem-solving activities [18]. In undergraduate nursing education, the flipped classroom has been described as a structured pedagogical approach organised around pre-class, in-class, and post-class components, rather than merely providing students with learning materials in advance. Pre-class learning commonly relies on online resources such as short video lectures, narrated presentations, readings, and quizzes, whereas classroom time is reoriented toward active, student-centered work focused on applying and analyzing prior learning, often through small-group activities and teacher clarification of difficult concepts [19].

This approach has demonstrated notable advantages in the academic performance of students pursuing health science degrees. A meta-analysis of 44 studies involving 7813 students showed a moderate positive effect on examination grades and results compared with traditional lecture-based classes. The standardized mean difference was recorded at 0.57 across disciplines such as medicine, nursing, and pharmacy [20]. At a broader level, evidence syntheses in nursing education indicate that flipped classroom interventions are generally associated with improved academic performance and favorable evaluations by students and faculty, although the available evidence remains methodologically heterogeneous and further high-quality studies are still needed [21]. Furthermore, it has been shown to be an efficient and adaptable strategy suitable for various educational contexts, encompassing both in-person and online modalities, thereby reinforcing its applicability within hybrid learning environments [22].

However, when the focus shifts specifically to EBP the available literature remains limited. The adoption of this teaching model has been shown to be effective in enhancing undergraduate nursing students’ attitudes, skills, and overall EBP competence compared with conventional teaching methods [23]. A mixed-methods study on evidence-based nursing learning through case-based and flipped learning reported perceived benefits in knowledge integration and transfer, teamwork, communication, and critical thinking, while also identifying difficulties and challenges related to the high time demands of the method, increased pre-class workload, limited time for in-class discussion, and difficulties associated with the length and suitability of the nursing cases used [24].

Qualitative research has demonstrated numerous positive experiences and perceived advantages among nursing students engaged in the flipped classroom model [25,26,27,28,29]. These benefits encompass cognitive, affective, and social dimensions of learning. This methodology has been observed to encourage an active, participatory environment, enhance interest in learning, foster autonomy, and strengthen critical thinking and problem-solving skills [25,26]. In addition, it promotes teamwork and the development of communication skills, enriches the acquisition of theoretical and practical knowledge, and improves the integration of clinical content into nursing practice [25]. Conversely, it presents challenges such as work overload, limited self-discipline, and difficulties in fully substituting the traditional approach [28]. Recent qualitative studies in nursing have examined students’ experiences of flipped classroom in broader undergraduate nursing settings or in non-EBP subjects, such as core nursing courses and paediatric nursing, rather than specifically within an EBP-focused course [25,26]. In addition, a recent systematic review highlighted the relevance of analyzing the flipped classroom from the student perspective and concluded that several factors that influence students’ experiences of this methodology are shaped by several interrelated factors, including the degree of structure in the learning design, the complementarity between off-campus and in-class activities, the use and balance of digital tools, the quality of group interaction, and the challenges associated with adapting to a new learning strategy, independent study workload, and time organisation [28].

The application of the flipped classroom remains a challenge in nursing university training, and the available literature provides limited evidence about its specific use in teaching EBP [26]. Qualitative studies show that this model, when applied to EBP, allows transferring learning to the home through technology, freeing up time in the classroom for interactive activities and fostering the independent learning of basic concepts by the student, dedicating the classroom hours to critical thinking, problem resolution, and the practical application of EBP [24]. Many authors agree that although the model has significant potential to promote student engagement and support meaningful learning, further research is necessary to optimize its pedagogical application [26,28]. Additionally, the model presents challenges related to time management and student fatigue, which need to be thoroughly analyzed across different contexts [24]. By focusing on students’ perceptions and experiences within an EBP-focused course, the present study seeks to address this gap and to extend current knowledge on how the flipped classroom supports the development of EBP competence and how barriers and facilitators shape that learning process. Therefore, this study aimed to explore, using a qualitative approach, the perceptions and experiences of nursing students enrolled in an Evidence-Based Practice course delivered through a flipped classroom model, examining their EBP learning processes, competency development, and critical thinking, as well as their direct experience of the blended instructional design and the perceived facilitators and barriers within the educational and clinical setting.

## 2. Materials and Methods

### 2.1. Design

The study was conducted within an interpretive-constructivist paradigm, based on the methodological framework proposed by Holloway and Galvin [30]. This epistemological stance assumes that participants’ experiences in learning EBP are socially and contextually constructed, and that the researcher’s role is to access and interpret the meanings they attribute to those experiences. Holloway and Galvin [30] situate this approach within an interpretive tradition of qualitative research in the healthcare field, emphasizing researcher reflexivity and the coherence between epistemological assumptions and methodological decisions.

This approach is particularly suitable for studying complex educational phenomena in naturalistic settings, as it prioritizes participants’ subjective perspectives without imposing predetermined theoretical frameworks. The analysis was carried out using the reflexive thematic [31], which explicitly positions the researcher as an active co-constructor of meaning rather than as a neutral analyst. The reflexive and interpretive nature of this process was maintained throughout all analytical phases, including ongoing dialogue between researchers, note-taking, and iterative review of analytical decisions. The reporting of this study was guided by the Consolidated Criteria for Reporting Qualitative Research (COREQ), a 32-item checklist for qualitative research.

### 2.2. Setting and Participants

The study was carried out at the Faculty of Nursing at the University of Murcia during the 2024/2025 academic year. The study population consisted of students enrolled in the nursing degree course Evidence-Based Practice (EBP), which was delivered through a flipped classroom model utilizing an online platform. The nursing degree in Spain spans four academic years. The curriculum was revised in the 2024/2025 year, and during the study period, the EBP course was taught simultaneously in the 2nd and 4th years, as it was part of two different curricula. During the study period, the total number of students enrolled in the EBP course was 380, including 180 students in the 2nd year and 200 students in the 4th year.

### 2.3. Sample Selection

The inclusion criteria were having completed the EBP course in the 2nd or 4th academic year and being available to participate in the study. No students from academic mobility programs (e.g., Erasmus or SICUE) were included in the final sample. Therefore, all participants had followed their academic training within the home institution in relation to the course context. Students who had enrolled in the course a second time were excluded.

Given the accessibility of the Evidence-Based Practice (EBP) course to all students, purposive sampling was conducted between April and May 2025. Students were invited to participate voluntarily through an announcement in class, and those who expressed interest contacted the research team to arrange interviews. The number of students who explicitly declined participation was not documented, as recruitment was based on voluntary involvement responses. The sampling considered heterogeneity criteria to obtain the maximum variation according to: gender, age, 2nd or 4th academic year, exam session in which the course was passed, employment, level of education, and previous EBP training. The final sample size was determined based on the principle of information power [32], according to which the sample size required in qualitative research depends on the informational density of the data in relation to the study aim, the specificity of the sample, the quality of the dialogue in the interviews, and the analytical strategy employed. Given the specific aim of the research, the use of purposive maximum variation sampling across key dimensions (academic year, prior training, employment status, and examination sitting), the depth and richness of the interview accounts, and the interpretive nature of reflexive thematic analysis, 20 participants were considered to provide sufficient information power to address the research question. The decision to conclude recruitment was made when, upon periodic review by the researchers, the thematic categories had achieved sufficient internal coherence and were conceptually robust enough to generate a credible and well-grounded interpretation of the phenomenon under study.

### 2.4. Design and Implementation of EBP Training

The EBP course has a load of 6 ECTS (European Credit Transfer System) and is taught during the first trimester (September–December) over a 15-week period, totaling 150 h. Of these hours, 60 were in-person, and 90 were for the student’s independent work.

The EBP course is taught by 5 professors with specific EBP training and more than 15 years of experience in this area. The students must complete 30 h of theory, 5 h of seminars, and 25 h of laboratories. The differences among the types of teaching are rooted in the degree of instrumentality and supervision required to acquire the competency under study. Thus, the students are organized into groups of 80 for the theoretical classes, 45–50 for the seminars, and 18 for the laboratories.

Before attending the in-person seminars and laboratories, students accessed the online learning platform, e-Toolkit, for pre-class asynchronous activities, as outlined in Table 1.

The platform is organized into seven open-access modules that require prior registration and are available at: https://europeannursingebp.com/. These modules align with the seven EBP steps described by Melnyk et al. [33]. Each module’s content was created using a variety of multimedia materials, including videos, clinical scenarios, questionnaires, and interactive games, to facilitate student self-learning. At the end of each module, students completed a test to assess their understanding of the content. During in-person classes, instructors reviewed key concepts, provided feedback, and supervised students’ work. A detailed description is available in a previous study [34].

In Module 0, after 2 h of asynchronous platform work, students participated in a theory session (3 h) consisting of structured group debate on the concept of EBP, its advantages and disadvantages, and the review of clinical variability examples. Module 1 required 3 h of prior asynchronous work, followed by a theory session (2 h) and a seminar (2.5 h) in which students formulated PICOT questions based on clinical scenarios and discussed their clinical domain. Module 2 involved 6 h of pre-class platform activities, followed by a seminar (2.5 h) and a laboratory (5 h) focused on evidence searching, with hands-on practice using the Cochrane Library and PubMed and group analysis of findings. Module 3 was the most extensive component, with 14 h of asynchronous preparation. In-class sessions included theory covering study types and data interpretation (6 h), common sources of bias in primary and secondary studies (2 h), and quality appraisal systems, levels of evidence, and grading of recommendations including the GRADE system (3 h). Each study design—cross-sectional analytical studies and diagnostic tests, case–control, cohort, randomized clinical trial, and systematic review—was further addressed in a dedicated theory session (2 h) paired with a laboratory (2.5 h) in which students pre-read an article and engaged in small-group critical appraisal using the CASP checklist. Modules 4 and 5, each preceded by 2 h of asynchronous work, were addressed jointly in a theory session (3 h) devoted to group discussion on the implementation of Clinical Practice Guidelines, including facilitating factors, barriers, and implications for clinical decision-making. Finally, Module 6 involved 1 h of pre-class platform work, followed by a theory session (1 h) and an extended laboratory (7.5 h) devoted to group preparatory work and formal oral presentation of results in response to a clinical question. Throughout the course, students also carried out independent and team-based work outside the classroom to develop the group project presented in this final session, applying the competencies acquired across all previous modules to address a real clinical question through the full EBP process.

All pre-class activities were delivered asynchronously via the digital platform. In-person sessions were led by nursing faculty with expertise in EBP and research methodology, who deepened key concepts, provided individualized and group feedback, and facilitated active learning techniques including structured debate, PICO question formulation, real-time database searches, critical appraisal exercises, and team-based oral presentations.

### 2.5. Data Collection

Sociodemographic variables were recorded: age, sex (female, male), previous level of education (none, technical school, other degree or Master’s), current academic year (2nd, 4th), employed while studying (yes, no), exam session in which the course was passed (December, May, or June), or if the course had not been passed yet.

The information was gathered through semi-structured interviews, designed to allow flexible exploration of participants’ experiences. A pre-developed interview script served as a reference framework, covering all main topics of the study while enabling questions to be adjusted dynamically based on the answers and the context of each participant. The interview guide explored the following main topics: participants’ prior knowledge of and expectations about EBP; previous exposure to EBP in other courses and clinical placements; experiences with the flipped classroom model and the online EBP e-Toolkit modules; perceived differences between autonomous online learning and face-to-face sessions; perceived effects of the course on attitudes toward EBP, knowledge, and skills; suggestions for improving the online and blended learning components; and the perceived applicability of EBP to theoretical learning and clinical practice, including barriers and facilitators for its use. To improve methodological transparency, the full semi-structured interview guide is provided as Appendix A.

In July 2025, the synchronous interviews were conducted via Zoom. These interviews were scheduled and mutually agreed upon with each participant to ensure their availability and a stable internet connection. The interviews were carried out by two research team members, VPM and AJRM, both of whom have research experience.

### 2.6. Data Analysis

The interview content was transcribed verbatim and subsequently analysed using reflexive thematic analysis [35], with the support of ATLAS.ti software (version 25.0.1 (32922)) for data management and code organisation. The analytical process followed the six-phase framework proposed by Braun and Clarke (2006) [31], adapted in accordance with their reflexive approach [36]. Phase 1 (Familiarisation with the data) involved an exhaustive and independent reading of all transcripts by both researchers (AMR, VPM) prior to initiating coding, with the aim of achieving in-depth familiarity with the data and beginning to identify emergent patterns of meaning; analytical memos were written throughout this phase to record initial impressions and potential interpretive directions. Phase 2 (Generating initial codes) was conducted inductively and close to the data, preserving participants’ voices and avoiding the premature emergence of predefined conceptual categories; each researcher independently coded the transcripts, producing detailed analytical memos to document coding decisions and emerging interpretations. Phase 3 (Searching for themes) involved grouping codes into candidate themes using visual thematic maps, followed by discussion sessions to compare independent codings, resolve disagreements, and identify convergent and divergent patterns. Phase 4 (Reviewing themes) entailed systematic checking of candidate themes against the full dataset to verify their internal coherence and their relevance to the research question; themes that were insufficiently grounded in the data or that showed conceptual overlap were merged, subdivided, or discarded. Phase 5 (Defining and naming themes) was carried out collaboratively through discussion and consensus. Phase 6 (Producing the report) involved the selection of representative verbatim extracts to illustrate each theme and subtheme. Throughout the entire analytical process, both researchers maintained a reflexive journal documenting their prior perspectives, epistemological assumptions, and the potential influence of these on analytical decisions, in accordance with the standards for rigour in reflexive thematic analysis.

### 2.7. Ethical Compliance

The study was approved by the Ethics Committee of the University of Murcia (256/2018). All procedures adhered to the ethics guidelines outlined in the Declaration of Helsinki. Student participation was voluntary, following an explanation of the study’s purpose and ethical safeguards. Student anonymity was preserved, and data confidentiality was ensured using a personal code.

## 3. Results

A total of 20 interviews were conducted. The average duration was about 40 min. The sample was mostly female, with ages ranging from 20 to 57 years old, and consisted of 2nd and 4th-year students who had passed the course in different exam sessions. Many participants studied and worked, and several mentioned previous degrees. The sociodemographic and academic characteristics of the interviewees are detailed in Table 2.

From the thematic analysis of the interviews, three large categories were identified that described the process of formative and professional transformation of the students with respect to Evidence-Based Practice (EBP), experienced within the specific pedagogical context of the flipped classroom model. While Categories 1 and 2 predominantly reflect the students’ engagement with EBP as a content domain, including attitudinal, cognitive, and identity-related transformations, Category 3 more directly captures their experience of the blended instructional design itself. Together, the three categories illustrate how learning EBP transcended the acquisition of theoretical content, favoring cognitive, attitudinal, and professional transformation that unfolded within and was shaped by a blended learning environment.

Below, each identified category and subcategory is described in more detail. Additionally, an expanded table (Appendix B) is included, presenting the most representative verbatim text for each category–subcategory.

1. Transforming the meaning of learning EBP and the professional role
  This first category captures how students’ relationship with EBP, as both a body of knowledge and a professional practice, was progressively redefined throughout the course. It encompasses three interrelated processes: an initial transformation from apprehension and fear of difficulty towards a genuine recognition of the practical usefulness of EBP (subcategory 1.1); a shift in attitude characterised by greater intellectual curiosity, critical engagement, and a personal commitment to ongoing learning (subcategory 1.2); and the gradual incorporation of EBP as a constitutive element of nursing professional identity, understood not as an external academic requirement but as the ethical foundation of evidence-informed care (subcategory 1.3). Although the accounts in this category primarily reflect students’ relationship with EBP as a knowledge domain, it should be noted that this formative transformation unfolded entirely within the context of the flipped classroom model, which, through its structured alternation of autonomous online preparation and active in-person sessions, created the conditions for this progressive appropriation of EBP.
  1.1. From initial fear to discovering usefulness
    The discourses revealed a significant shift in expectations regarding the EBP course. Over time, it was observed that these expectations evolved, and that students’ views of learning EBP and their professional identity were transformed. Before taking the course, a predominantly negative expectation existed, not centered on understanding the content but on how difficult it would be to pass. This shows that initial expectations were more strongly shaped by peer discussions, especially among those in higher academic years, than by direct experience. This negative expectation stemmed from insecurity and fear: “*they really scared me about the subject*,” (E13); “*everyone was saying it was really hard*,” (E20).
    The initial perception changed as the course progressed, evolving into an awareness of its usefulness, relevance, and practical value. “*It has helped me a lot because now I have more knowledge and I know where to look for information*” (E6). The students recognized that it provided them with solid tools for their training and future careers. “*Once you study it, you know it’s very useful and that it will be very useful to you in the future*,” (E5). This shift from fear of difficulty to recognition of usefulness suggests an early epistemic reorientation: EBP began to be understood not as an abstract or burdensome academic subject, but as a meaningful way of approaching professional knowledge and action.
  1.2. Changes in attitude and commitment to knowledge
    The formative experience fosters a more active, reflective, and responsible attitude toward learning and the nursing profession. On one hand, students recognize the practical value of EBP: “*It’s true that this subject has made me ask myself more questions*” (E1); “*I didn’t use to pay attention to it, but now when I don’t know something, I look for and compare information*” (E2).
    Many participants realized that they had previously viewed the course as just an academic requirement, but after completing it, they experienced a rise in interest, motivation, and enjoyment of learning: “*Now I see the importance of staying up-to-date, because if you’re not, you can harm the patient without realizing it*” (E6).
    The attitudinal change was also reflected in the willingness to continue learning independently beyond the course: “*What has changed me the most is that now I’m interested to keep learning*” (E20); “*I’ve learned to differentiate between what is reliable and what is not*” (E14). These accounts point to a change not only in motivation, but also in students’ relationship with knowledge itself: information was no longer received passively, but evaluated, contrasted, and linked to responsibility in care. In this sense, learning EBP appeared to promote a more questioning stance toward knowledge, consistent with the development of critical thinking.
  1.3. Evidence-Based Practice as a hallmark of professional identity
    The discourses showed that learning EBP transcended the academic sphere and was integrated as part of the professional identity under construction, strengthening the ethical sense and professional responsibility: “*If nurses are trained and up-to-date, they provide better care*” (E6); “*Doing something knowing why we do it, or why we know it’s right*” (E1).
    Many students expressed that EBP defined what it means to be a nurse: “*Ultimately, everything you do has to have a reason, because it also has consequences*” (E19); “*Our daily practice in the hospital is based on this, on studies that people have done to see which technique is best*” (E17). From this perspective, EBP was not described as an external requirement added to nursing practice, but as a constitutive element of professional identity. The students’ accounts suggest that learning to justify care with evidence contributed to shaping an image of the nurse as a reflective, accountable, and ethically grounded professional.
2. Cognitive and metacognitive processes in the learning of EBP
  This second category describes the internal cognitive and metacognitive changes that accompanied students’ engagement with EBP throughout the course. Three dimensions were identified: the development of critical thinking as an active, questioning stance towards established practice and received knowledge, moving beyond the mere repetition of procedures towards analytical reasoning about clinical decisions (subcategory 2.1); the emergence of meta-learning and professional awareness, understood as a reflective consciousness of how knowledge is produced, validated, and applied, which translated into a commitment to continuous updating as a professional responsibility (subcategory 2.2); and the adoption of expansive and self-regulated learning strategies, whereby students autonomously sought out additional resources and managed their own learning pace (subcategory 2.3). While subcategories 2.1 and 2.2 emerge primarily from students’ engagement with EBP as a content domain, subcategory 2.3 most clearly reflects the structural affordances of the flipped classroom model: the open-access, modular design of the e-Toolkit platform directly enabled students to organize their own study timing and pace, supporting the development of self-regulated learning behaviours that the flipped design was specifically structured to promote.
  2.1. Learn how to think critically and with sound reasoning
    The course marks a point of cognitive turning, where students learn to question what they previously accepted without question: “*Before, you did things because you were told to, but now you think: why is it done this way? Is there a better way?*” (E1); “*I often think about things and get curious, wondering: Is this really well done?*” (E16). Critical thinking becomes a tool for making informed decisions, breaking the cycle of routine logic: “*When someone tells you,* “*It’s always been done this way,*” *and you respond,* “*Yes, yes, it’s always been done this way,*” *that works verbally. Maybe further study is needed to explain why it’s done that way*” (E20); “*When I don’t know something, I look it up so I can do it properly and understand why I’m doing it*” (E10). These narratives suggest that students began to see critical thinking not only as an academic skill, but as a way of questioning routine practice and understanding why care is provided in a certain way. Rather than simply repeating procedures, they described a more reflective and analytical approach to clinical decision-making.
  2.2. Meta-learning and professional awareness
    Beyond technical knowledge, the students developed a reflective awareness of learning and its impact on clinical practice: “*If we stick to how things were done 50 years ago, we may be harming the patient*” (E2); “*Evidence-based practice is a tool that every nurse should use to provide appropriate patient care*” (E4).
    Becoming aware is translated into a commitment with constant updating and continuous learning: “*I know we always have to stay up-to-date; if we don’t know something, we have to go to the evidence*” (E6). For many students, this understanding represents an early professional maturity, a way to “learn how to learn” that transcends the course and is projected onto their future professional practice. At this level, the findings reflect a transformation in how students understand knowledge and learning: they do not merely accumulate information, but become more aware of how knowledge is produced, validated, and applied in practice. This meta-learning dimension helps explain why EBP was associated with a more mature and self-conscious professional stance.
  2.3. Expansive and self-regulated learning
    EBP promotes independent and multimodal learning strategies. With respect to expansive learning, the participants frequently mentioned the use of complementary digital resources: “*I watched videos on my own on YouTube and all sorts of things*” (E15); “*on TikTok of a nurse talking about evidence-based practice*” (E1).
    With respect to self-regulated learning, they underlined the ability to manage one’s own timing and pace through the online learning platform used in the course: “*You could organize your work and review the modules whenever you wanted*” (E12); “*The best thing is having the information at hand and being able to review it*” (E17). This self-regulated use of multiple resources suggests that students were not limited to following the course requirements, but were progressively assuming a more autonomous role in the management of their own learning. Such autonomy is relevant because it reinforces the idea of EBP learning as an active and sustained process that extends beyond the course context.
3. The formative experience as a catalyzer of deep learning
  This third category is the most directly grounded in students’ experience of the flipped classroom model as a pedagogical format and constitutes the section of the findings that most explicitly addresses the instructional dimension of the study aim. Three dimensions were identified: the blended model as a space for active knowledge construction, in which the combination of pre-class asynchronous preparation via the e-Toolkit and in-person active sessions enabled students to arrive with a prior foundation and engage more deeply with content during face-to-face time (subcategory 3.1); clinical practice as a setting for confrontation and transference, where 4th-year students tested their EBP learning against the realities of clinical environments, negotiating tensions between evidence, tradition, and clinical experience (subcategory 3.2); and the facilitators and barriers of the practical context, including the role of evidence-oriented clinical supervisors and the structural and cultural constraints, such as care overload and resistance to change among more senior professionals, that shaped the extent to which EBP could be applied in practice (subcategory 3.3).
  3.1. The blended model as a space of active construction
    Students particularly appreciated the blended learning model or the flipped classroom, which combines independent study with in-person instruction, as it allows a connection between autonomy and teacher support. The students underlined the value of previously accessing the materials from the online platform EBP eToolkit before the in-person classes: “*But I think this method is more… it makes the process a bit easier, because you go to class and they explain the content you’ve already read. I find that much easier. Also, if everyone reads it at home, it speeds up the class for the professor a lot. It becomes more dynamic, faster*” (E5). This approach allowed them to arrive to class with previous background and make better use of collaborative work: “*Even if you think you haven’t grasped certain concepts, they’re explained in class, and since you already have a foundation, it makes understanding much easier*” (E2). The digital platform is associated with autonomy and flexibility, as it provides them with the possibility of managing the pace and timing of their learning: “*It gives you freedom and autonomy; it’s a resource that’s always there*” (E10)
    The in-person classes were valued for their capacity to contextualize, provide practical examples, and resolve doubts, giving a practical sense to the concepts that were learned independently: “*I prefer having someone there to explain it to me, perhaps with real-life examples or their own experience*” (E1); “*It’s best to have the teacher there to answer questions and help you understand the concepts*” (E2).
    Many expressed that this format increases their motivation and involvement, as it forces them to prepare and actively interact: “*It’s not just about going to class, getting all dressed up, and that’s it, listening; I think that also motivates you, and that’s why you learn more*” (E1). These accounts indicate that the blended model functioned as a pedagogical space for active knowledge construction, in which prior exposure to content and subsequent classroom interaction facilitated deeper engagement with EBP. Rather than simply improving access to materials, the model appeared to modify how students participated in learning and how they connected independent preparation with classroom-based clarification and application.
    Nevertheless, they pointed to some aspects that could be improved, such as the excessive length of some modules: “*Some topics were very long. Perhaps being more concise, more specific, would be better.*” (E2), and the need to include more audiovisual resources: “*Some short explanatory videos of the content would be needed*” (E9).
  3.2. Clinical practice as a setting for confrontation and transference
    During the clinical practices, the 4th-year students who were conducting practices in the clinical context at the same time that they were taking the course, tested the EBP, which became a criterion of personal reflection. The students identified discrepancies between what was learned and what they observed in the reality of care: “*A nurse told me one thing, but the evidence I found was different*” (E4). However, the process of confrontation was not solely experienced as conflict, but also as progressive integration. The students learned to recognize the complexity of the reality of care and to reflect on the reasons behind the clinical actions: “*The younger ones look for evidence; the older ones say it’s always been done this way*” (E18); “*When I asked, they told me: it’s the same whether you put the cap on or not if you clean it beforehand, and then I verified that it was true in the evidence*” (E19). This tension between what was taught and what was observed in practice seems to have acted as a catalyst for deeper learning. It placed students in an interpretive position in which they had to negotiate between tradition, clinical experience, and evidence, thereby reinforcing both critical judgment and the emerging sense of professional responsibility.
  3.3. Facilitators and barriers in the practical context
    The students who completed their internship practices in environments in which the tutors showed an open and up-to-date attitude described very positive learning experiences: “*I met a tutor… who often showed me, whenever I had any question, how she looked for information… what databases she used and did it right then and there during the exam, and everything. She answered any question I had and mentioned the importance of looking for evidence in practice*” (E14). Other participants underlined the importance of collaborative work and the learning environment in healthcare units: “*For example, during my dialysis rotations, especially when patients asked about what they could and couldn’t eat, we definitely looked for information about that. We researched thoroughly to give them accurate information about what they could and couldn’t consume*” (E6). Mainly in more complex units such as intensive care: “*The area where I saw the use of evidence most clearly reflected in practice was in the ICU, because there were so many specific operations… I really saw that there was much more practice in seeking information and staying up to date*” (E4).
    On the other hand, many students described environments that were reluctant to change, in which the practice was based on tradition or the authority of experience, associated with more senior health professionals: “*But it’s true that there are others who do things the old-fashioned way. What they’ve learned and what they’ve seen and so on*” (E16). “*I’ve met older people, and they’re more traditional, they don’t look for the reason or the cause, but maybe they ask a colleague, who’s the one who knows, but they don’t look for evidence*” (E18); “*It was like it was a tradition. I mean, this is how it’s done because it’s always been done this way*” (E18).
    They also mentioned structural barriers related to care overload and the lack of resources, which, from their point of view, justified the scarce use of EBP in clinical practice: “*It’s also true that the resources aren’t there either… So you can’t use it either. I mean, sometimes it’s not the professionals. They simply don’t have other resources*” (E2). These findings show that the development of EBP competence was not perceived as depending exclusively on individual motivation or training, but also on the practice culture in which students were socialized. In this regard, clinical placements functioned as spaces where professional identity could be either reinforced through evidence-oriented role models or constrained by organizational and cultural barriers that normalized routine-based care.

## 4. Discussion

The findings from this qualitative study indicate that the experience of acquiring evidence-based practice (EBP) skills through a flipped classroom methodology in the nursing program was a distinctly transformative process. The students described a journey that ranged from initial fear and viewing the course as difficult and disconnected from nursing practice to recognizing its usefulness in promoting care and “doing what is right” in terms of safety and quality, thereby enhancing nursing students’ ability to provide evidence-based care [37]. This progression aligns with previous evidence indicating that EBP training programs significantly improve nursing students’ knowledge, skills, attitudes, and overall EBP competence [11,34,38]. In this regard, acquiring EBP competence relies not solely on technical skills such as literature searching, but on active pedagogical approaches designed to foster critical thinking and student ownership of their learning. Indeed, evidence indicates that traditional didactic models are often limited and can lead to student disengagement when addressing complex EBP concepts. Therefore, implementing innovative and interactive teaching strategies is essential to adequately prepare nurses to integrate evidence into their clinical decision-making [39].

Initially, students indicated that their negative view of the EBP course was not due to a lack of knowledge about the content but rather to a socially constructed expectation of difficulty. After gaining experience, this initial perception changed, and EBP was seen as an essential tool for their future careers and as a foundation for care. Our findings align with those of others [40], who used Rogers’ innovation diffusion model to organize students’ experiences into the stages of knowledge, persuasion, decision, implementation, and confirmation of EBP, illustrating a gradual process of acceptance and future use. Additionally, research and EBP training have been described as an ambivalent experience, simultaneously perceived as challenging and burdensome, yet also useful and relevant for future professional practice, which aligns with the initial perception of difficulty and usefulness observed in our study [41].

This attitudinal change is closely linked to the construction of professional identity. The students even described EBP as a trait that defines “what it means to be a nurse,” as it involves justifying interventions and continuously updating knowledge. This aspect aligns with evidence [42], which characterized nurse professionalism as a dynamic concept supported by professional education, a reliable and constantly updated clinical competence, and a clear commitment to quality of care and responsibility in practice.

Regarding the cognitive and metacognitive changes from learning EBP, the students described the course as a key turning point in their thinking: they no longer accepted clinical practices uncritically but started to ask questions, challenge routine logic, and actively seek evidence to support their decisions. This development of critical thinking aligns with existing research, which shows that EBP training programs can significantly improve EBP skills, critical thinking, and problem-solving abilities [11].

Additionally, the results show a clear element of meta-learning. The students can incorporate an awareness of the importance of continuous updating into their clinical practice, recognize the risks of relying on outdated methods, and remain self-aware of their responsibility to seek out and assess evidence when doubts arise. These findings align with previous studies that suggest that using research is critical for nursing students to stay current, improve problem-solving skills, and make autonomous decisions in clinical settings [43].

In our study, students described the combined use of scientific databases (such as PubMed, Scopus, or Cochrane) and institutional resources available in health centers (infographics, protocols, and clinical guides), along with informal digital resources like YouTube and TikTok, which indicates a multimodal learning pattern characteristic of the new generations. This approach aligns with studies showing that nursing students coordinate academic resources and digital social networks to enhance and broaden their learning [44,45]. From an educational standpoint, this diversity of resources underscores the importance of explicitly including information literacy skills in EBP training, which is recognized as a core component of EBP competency in nursing students [46].

The third set of ideas examines the evaluation of the blended learning model based on the flipped classroom, supported by the EBP e-Toolkit platform. Students found the combination of prior independent work with online materials and in-person sessions focused on clarifying doubts, discussing, and applying clinical cases to be particularly helpful. This evaluation aligns with previous research indicating that the flipped classroom model supported by the EBP e-Toolkit is effective for teaching EBP and is linked to significant improvements in students’ attitudes, skills, and overall EBP competency, with no differences observed compared to traditional teaching in the knowledge dimension [23].

Compared with traditional lecture-based teaching, the experiences described by our participants suggest a more active role for students in learning, greater preparation before class, and more explicit opportunities to discuss and apply EBP-related content during face-to-face sessions. The participants emphasized that prior access to the content helped them arrive at class with a conceptual background, follow explanations more effectively, and use the in-person sessions to explore topics more deeply. This view aligns with studies that examined the combination of the flipped classroom and problem-based learning in a Pediatrics course, which found increased interest, motivation, independent learning, and teamwork among students, as well as a higher perceived workload [25,47].

Beyond students’ positive evaluation of the blended format, our findings suggest that the specific contribution of the flipped classroom component lies in enabling students to engage with content before class and to use face-to-face sessions for discussion and application. This interpretation is consistent with previous studies showing that flipped classroom approaches in nursing education are organised around pre-class preparation and active in-class learning, and are generally associated with favourable learning experiences and outcomes [21,24,28]. From this perspective, the value of the model lies not only in combining online and face-to-face teaching, but in promoting a more active and prepared engagement with EBP learning [21,24].

At the same time, our findings also align with those reported in other active learning approaches, such as case-based and problem-based learning, which have been associated with greater engagement, teamwork, and deeper involvement in the learning process [24,25]. In this sense, the distinctive contribution of the present study lies in showing how these experiences are articulated within an EBP-focused course delivered through a flipped classroom-based blended model supported by the EBP e-Toolkit.

The EBP e-Toolkit online platform was seen as a resource that promotes autonomy, flexibility, and the regular review of content. This positive view coexists with the recognition of areas for improvement, such as lengthy modules, too much text, and a need for more videos and feedback opportunities. These aspects that are coherent with evidence recommending short-term EBP programs supported by active and blended teaching methods [24]. Furthermore, the utilization of the platform as a space for autonomous work aligns with evidence demonstrating that interactive methodologies in virtual environments facilitate the development of applied knowledge and competencies such as independent learning, critical thinking, and teamwork among nursing students [48].

The experience in a clinical practice setting emerges as a key scenario in which learning about EBP is tested and consolidated. The students described the confrontation between the evidence found and the practices observed, especially when specific health professionals justified their actions with the argument of “it has always been done this way”. This logic of habit was identified as an obstacle against the evolution of the profession and the implementation of EBP, to the point of proposing the banishment of “it has always been done this way” from the minds of nursing professionals to make advances towards evidence-based care [49,50]. These findings are related to that described as a theory–practice gap in the use of research by nursing students [43].

Another important point to consider when examining clinical practices as key spaces is that students who described units with coherence between what they learned at university and what they observed during the service also mentioned receiving support from tutors, finding real opportunities to apply their knowledge, and experiencing practices as worthwhile training experiences. Similarly, other studies indicate that complex clinical environments can either hinder learning or promote it, depending on the support and supervision students receive [51].

Although the study was not designed as a comparative analysis between participant subgroups, no clearly differentiated patterns emerged across most sociodemographic or academic profiles. However, some contextual nuances were observed. In particular, fourth-year students, who were simultaneously engaged in clinical placements, were able to describe more directly the confrontation between what they learned in the course and what they observed in practice. This suggests that the opportunity to contrast EBP learning with real clinical contexts may shape the way students experience and interpret the course.

Our results showed that when students had tutors who sought evidence in real time, consulted protocols, and legitimized the question, EBP was naturally integrated into practice and perceived as attainable, as seen in students involved in EBP projects and knowledge translation. Conversely, in environments characterized by healthcare overload, resource scarcity, and a culture based on “it has always been done this way,” students viewed EBP as a challenging yet desirable goal, reproducing known barriers related to organizational culture and the gap between theory and practice in research use.

The integration of our results with existing knowledge enables us to draw many educational implications. Firstly, the findings reinforce the importance of incorporating the EBP course into the nursing curriculum, as it shifts initial attitudes of rejection or insecurity about the content and its applicability, and establishes evidence as the foundation of practice. This helps develop a professional identity focused on well-founded, reflective, ethical, and high-quality care. Such integration is a crucial step toward overcoming the barriers discussed in the literature and laying a strong educational foundation [52].

In second place, the data support the use of blended learning models that combine structured digital platforms, the flipped classroom, and in-person activities focused on application, consistent with evidence showing that active and multimodal methods, including the flipped classroom, achieve similar or better student satisfaction than traditional teaching [11,21,23,25,38]. To effectively integrate EBP, nursing educators should move beyond isolated research assignments and instead embed EBP principles within clinical simulations and laboratory work. Practical recommendations include the use of ‘EBP-focused’ sections for every clinical topic covered and the implementation of interactive strategies, such as the flipped classroom, to enhance student engagement [39].

In third place, the results emphasize the importance of involving clinical settings in EBP training. The presence of a clinical tutor trained in evidence-based practice, the availability of current protocols, and a unit environment that encourages questioning and seeking information are identified as key elements to ensure that EBP remains more than just a theoretical concept [51,53,54]. The present study offers a qualitative and in-depth understanding of how EBP training impacts nursing students, complementing previous quantitative findings on the effectiveness of educational interventions in undergraduate programs [11,23,34].

### Limitations of the Study

The present study has several limitations that must be considered when interpreting the results. Firstly, participation was voluntary, which introduces the potential for self-selection bias, as individuals who consented to participate in interviews may have possessed a greater interest in EBP or experienced more positive (or more intense) engagements with the course. Furthermore, data collection was conducted via individual interviews, a method that may exacerbate social desirability bias, particularly if the interviewer is affiliated with the teaching staff, despite assurances of confidentiality and anonymity. To mitigate response bias and prevent any influence on students’ answers, the course instructors did not partake in the interviews. Finally, the study explored perceptions at a specific point after course completion; it did not facilitate the evaluation of the durability of attitudinal and cognitive modifications over time, nor their effective translation into clinical behaviors once individuals entered professional clinical settings. In addition, the study did not specifically explore the use of artificial intelligence tools in students’ autonomous learning, which may have limited a more comprehensive understanding of the digital resources currently involved in the development of EBP-related competencies. Future longitudinal investigations and the employment of mixed methodologies may supplement these findings and provide a more comprehensive understanding of the true influence of EBP training on clinical application.

## 5. Conclusions

The results of the qualitative study show that EBP training with the flipped classroom approach was experienced by students as a transformative process. The students described a journey from initial fear and perceiving difficulty to recognizing EBP as an essential tool for guiding decisions, challenging routines, and developing a professional identity.

Similarly, the study emphasizes the pivotal role of blended learning methodologies, which promote autonomous and adaptable working styles, complemented by instructor support and continuous engagement within the classroom setting. Furthermore, the significance of the clinical context was evidenced in EBP training, as it represents a real-world environment in which students encounter the concepts learned during the course and recognize the complexities involved in their practical application within healthcare.

Overall, the findings endorse the incorporation of EBP as a mandatory course within the curriculum, thereby enhancing critical thinking and information literacy competencies, and ensuring alignment between the university and clinical environments.

This study also contributes to the literature by providing qualitative evidence on how nursing students experience EBP learning within a flipped classroom-based blended approach, particularly regarding the development of critical thinking, engagement with evidence, and professional identity formation. Future research should examine the long-term sustainability of these changes, as well as their transfer to clinical practice across different academic and institutional contexts.

## Figures and Tables

**Table 1:** Flipped classroom programmed activities.

Pre-Class Asynchronous Activities	Duration	In-Class Synchronous Activities	Method (Duration)
Module 0: Cultivate a spirit of inquiry within an evidence-based practice (EBP) culture and environment	2 h	Discussion about the concept of EBP, advantages and disadvantages. Groups for and against. Review of clinical variability examples.	Theory (3 h)
Module 1: Ask the burning clinical question in PICOT format	3 h	Students pose PICOT questions based on clinical scenarios. They discuss the clinical domain of the questions.	Theory (2 h) Seminar (2.5 h)
Module 2: Search for and collect the most relevant best evidence	6 h	Search the Cochrane library. Perform searches using different search strings in PubMed. Analysis and discussion of the findings	Seminar (2.5 h) Laboratory (5 h)
Module 3: Critically appraise the evidence		Characteristics of the most important type of studies: cross-sectional, case and control, cohort, experimental, qualitative studies. Data interpretation. Discussion of data starting with studies published.	Theory (6 h)
		Description of the most common types of bias in primary and secondary studies.	Theory (2 h)
		Practical work of the evaluation systems of study quality, classification of the levels of evidence and degrees of recommendation. Practical example of the use of the GRADE system in clinical practice guidelines and systematic reviews.	Theory (3 h)
	14 h	Critical appraisal of cross-sectional analytical studies and diagnostic tests. Pre-reading of an article and class discussion in a small group about biases using the CASP checklist.	Theory (2 h) Laboratory (2.5 h)
		Critical appraisal of case and control studies. Pre-reading of an article and class discussion in small groups about biases using the CASP checklist.	Theory (2 h) Laboratory (2.5 h)
		Critical appraisal of cohort studies. Pre-reading of an article and class discussion in a small group about biases using the CASP checklist.	Theory (2 h) Laboratory (2.5 h)
		Critical appraisal of a randomized clinical trial. Pre-reading of an article and class discussion in a small group about biases using the CASP checklist.	Theory (2 h) Laboratory (2.5 h)
		Critical appraisal of a systematic review. Pre-reading of an article and class discussion in a small group about biases using the CASP checklist.	Theory (2 h) Laboratory (2.5 h)
Module 4: Integrate the best evidence with one’s clinical expertise and patient/family preferences	2 h	Group discussion on the implementation of a Clinical Practice Guideline. Review of factors in favor, barriers and implications in decision making in practice.	Theory (3 h)
Module 5: Evaluate outcomes of the practice decision or change based on evidence	2 h		
Module 6: Disseminate the outcomes of the EBP decision or change	1 h	Carrying out group preparatory work and group presentation. Presentation of results in response to a clinical question.	Theory (1 h) Laboratory (7.5 h)

**Table 2:** Characteristics of the interviewees.

Interviewee	Sex	Age	Academic Year of the Course	Exam Session When Course Was Passed	Work	Other Degrees	Interview Duration
1	Female	22	4th	December	NO	NO	53 min
2	Female	22	2nd	June	NO	NO	40 min 25 s
3	Male	21	4th	December	NO	NO	30 min 50 s
4	Female	24	4th	Pending	NO	NO	36 min 47 s
5	Female	21	2nd	June	YES	NO	38 min 11 s
6	Female	30	4th	June	YES	YES	34 min 48 s
7	Male	34	4th	Pending	NO	YES	39 min 42 s
8	Female	46	2nd	Pending	YES	YES	47 min 33 s
9	Female	20	2nd	June	NO	NO	34 min 51 s
10	Female	22	4th	December	YES	NO	29 min 04 s
11	Female	22	2nd	December	YES	YES	24 min 45 s
12	Female	21	2nd	May	NO	NO	36 min 50 s
13	Female	26	2nd	May	YES	YES	41 min 31 s
14	Female	22	4th	December	YES	NO	22 min 50 s
15	Female	20	2nd	May	YES	NO	23 min 48 s
16	Female	20	2nd	June	YES	NO	22 min 51 s
17	Female	23	4th	June	YES	NO	25 min 20 s
18	Male	25	4th	June	YES	YES	33 min 14 s
19	Female	20	2nd	December	YES	NO	37 min 15 s
20	Female	57	2nd	December	YES	YES	1h 41 min

## Data Availability

Data is unavailable due to privacy and ethical restrictions.

## Public Involvement Statement

No public involvement in any aspect of this research. All eligible nursing students were invited during class, and those who participated did so voluntarily.

## Guidelines and Standards Statement

This manuscript was drafted against the Consolidated Criteria for Reporting Qualitative Research (COREQ) [55] for qualitative research.

## Abbreviations

The following abbreviations are used in this manuscript:

EBP	Evidence Based Practice
ECTS	European Credit Transfer System
CASP	Critical Appraisal Skill Program

## Appendix A. Semi-Structured Interview Guide

**Interview Identification Data**

- Interview date:
- Interview location:
- Start time:
- Participant identification number:

**Introduction**

- Thank the participant for their availability and time.
- Explain the aim of the interview: to explore the participant’s expectations and experiences regarding the use and learning of Evidence-Based Practice (EBP) through the course completed within the Nursing Degree.

**Initial Questions**

- Please tell me your age:
- Sex:
- Current academic year:
- Did you pass the course in the first examination session?
- Do you work while studying? If so, how much time does it take?
- Do you have any previous qualifications? Which ones?
- Do you have any previous training in EBP?
- Is EBP an interesting topic for you?
- Before taking the course, did you know anything about EBP?
  ○ Please explain how it was addressed in other subjects.
  ○ Did you observe it during your clinical placements? If so, in what way?

**Main Section**
**Knowledge of EBP and Consolidation of Learning**

1. What relationship do you think exists between EBP and nursing?

**Experiences of Learning EBP through the Flipped Classroom**

1. Before taking the course, what idea did you have about it? Please describe your impressions before starting.
2. In this course, you studied EBP through a combination of traditional teaching and online learning, working on the course content at home using the EBP e-Toolkit modules. This content was then further developed during classroom sessions. Please tell me about your experience.
3. What did you think about autonomous learning through the online platform?
4. How did this autonomous learning through the online platform differ from face-to-face classes?
5. What did the online EBP course contribute to your learning?
  ○ Please mention three positive aspects.
  ○ Please mention three negative aspects.
6. What did the face-to-face component of the EBP course contribute to your learning?
  ○ Please mention three positive aspects.
7. Do you think that this flipped classroom teaching method leads to more learning than traditional teaching?
8. Do you think this training changed your attitude toward EBP?
9. Do you think this training improved your knowledge? To what extent? Can you give an example?
10. Did this training improve your EBP skills? Can you give an example?

**Suggestions for Improving the Online Training**

- I would like to know how the training you received could be improved. In what way could the course be made more user-friendly for future students?
  ○ Fewer hours?
  ○ Longer duration?
  ○ Changes in content?
  ○ Changes related to activities, tests, infographics, videos, or interactive presentations?

**Expectations and Determinants of the Applicability of EBP in Theoretical and Clinical Learning**

1. Describe situations from your clinical placements in which you observed the application of EBP in the work carried out by nursing professionals (e.g., your mentors or other professionals).
2. Describe situations in other nursing subjects in which EBP was used.
3. Explain how the knowledge and skills learned in EBP may help you when performing techniques during your clinical placements.
4. How could EBP be better learned during clinical placements?

**Closing**

- Is there anything else you would like to tell me that I should have asked about, even if I did not do so?
- Thank you for your time. Your testimony will be very valuable for our research.
- Please remember that, if you have any questions, you may contact us, and if you wish, we will inform you of the results of the study.
- End time:

## Appendix B

**Table A1:** Categories, sub-categories, and verbatim text.

Category	Sub-Categories	Verbatim Text
1. Transforming the meaning of EBP learning and the professional role	1.1. From initial fear to the discovery of usefulness	- “*they really scared me about the subject*” (E13). - “*everyone was saying it was really hard*” (E20). - “*It has helped me a lot because now I have more knowledge and I know where to look for information*” (E6). - “*Once you study it, you know it’s very useful and that it will be very useful to you in the future*” (E5).
	1.2. Change of attitude and commitment to knowledge	- “*It’s true that this subject has made me ask myself more questions*” (E1). - “*I didn’t used to pay attention to it, but now when I don’t know something, I look for and compare information*” (E2). - “*Now you have a little more knowledge that there is much more information that may be useful and that you may want to look for in it*” (E10). - “*What has changed me the most is that now I’m interested to keep learning*” (E20). - “*I’ve learned to differentiate between what is reliable and what is not*” (E14).
	1.3. Evidence-based professional identity	- “*If nurses are trained and up-to-date, they provide better care*” (E6). - “*Doing something knowing why we do it, or why we know it’s right*” (E1). - “*Ultimately, everything you do has to have a reason, because it also has consequences*” (E19). - “*Our daily practice in the hospital is based on this, on studies that people have done to see which technique is best*” (E17).
2. Cognitive and metacognitive processes in EBP learning	2.1. Learning to think critically and with sound reasoning	- “*I often think about things and get curious, wondering: Is this really well done?*” (E16). - “*Now I realize that whatever I’m about to do, I have to ask myself if what I’m doing is… I’m doing it correctly*” (E13).
	2.2. Meta-learning and professional awareness	- “*If we stick to how things were done 50 years ago, we may be harming the patient*” (E2). - “*Evidence-based practice is a tool that every nurse should use to provide appropriate patient care*” (E4). - “*I know we always have to stay up-to-date; if we don’t know something, we have to go to the evidence*” (E6).
	2.3. Expansive and self-regulated learning	- “*I watched videos on my own on YouTube and all sorts of things*” (E15). - “*on TikTok of a nurse talking about evidence-based practice*” (E1). - “*You could organize your work and review the modules whenever you wanted*” (E12). - “*The best thing is having the information at hand and being able to review it*” (E17).
3. The formative experience as a catalyst for deep learning	3.1. The mixed model as a space for active construction	- “*Well, I think it’s a good system, because that way you arrived in class not so lost. And also, since we were also doing exercises, this module app also came with exercises to do after finishing the topics, so I thought… I thought it was a very good idea for that, so you wouldn’t be so lost in class and could review at home.*” (E9). - “*Even if you think you haven’t grasped certain concepts, they’re explained in class, and since you already have a foundation, it makes understanding much easier*” (E2). - “*It gives you freedom and autonomy; it’s a resource that’s always there*” (E10). - “*It’s best to have the teacher there to answer questions and help you understand the concepts*” (E2). - “*Some topics were very long. Perhaps being more concise, more specific, would be better*” (E2). - “*Some short explanatory videos of the content would be needed*” (E9).
	3.2. Clinical practice as a setting for confrontation and transference	- “*A nurse told me one thing, but the evidence I found was different*” (E4). - “*The younger ones look for evidence; the older ones say it’s always been done this way*” (E18). - “*When I asked, they told me: it’s the same whether you put the cap on or not if you clean it beforehand, and then I verified that it was true in the evidence*” (E19).
	3.3. Facilitators and barriers of the practical context	- “*I met a tutor… who often showed me, whenever I had any question, how she looked for information… what databases she used and did it right then and there during the exam, and everything. She answered any question I had and mentioned the importance of looking for evidence in practice*” (E14). - “*The area where I saw the use of evidence most clearly reflected in practice was in the ICU, because there were so many specific operations… I really saw that there was much more practice in seeking information and staying up to date*” (E4). - “*But it’s true that there are others who do things the old-fashioned way. What they’ve learned and what they’ve seen and so on*” (E16). - “*It was like it was a tradition. I mean, this is how it’s done because it’s always been done this way*” (E18). - “*It’s also true that the resources aren’t there either… So you can’t use it either. I mean, sometimes it’s not the professionals. They simply don’t have other resources*” (E2). - “*(…) And that does not really happen in hospital wards. It is also true that the necessary resources are not always available. Many of the things that are available in primary care are not available in hospital settings. So, you cannot always apply them. In other words, sometimes it is not the professionals’ fault; they simply do not have other resources*” (E2).
